# Comparative Analysis of Maturation Prediction Methods (Moore, Mirwald, BAUSport^TM^): Croatian Female Volleyball Youth Team Example

**DOI:** 10.3390/jfmk10020171

**Published:** 2025-05-12

**Authors:** Drazen Cular, Tea Beslija, Marijana Cavala, Matej Babic, Ana Kezic

**Affiliations:** 1 Faculty of Kinesiology, University of Split, 21000 Split, Croatia; drazen.cular@kifst.eu (D.C.); marijana.cavala@kifst.eu (M.C.); matej.babic@kifst.eu (M.B.); 2 Einstein, Startup for Research, Development, Education, Trade and Services, 21000 Split, Croatia; 3 European Institute for Talents, Education, Research & Development, 21000 Split, Croatia; tea.beslija@hotmail.com; 4 Faculty of Kinesiology, University of Zagreb, 10000 Zagreb, Croatia

**Keywords:** somatic maturation, skeletal age, ultrasound technology, youth athletes, skeletal age, growth, talent identification

## Abstract

**Objectives**: The study aims to compare three distinct protocols—Moore, Mirwald, and the new BAUSport^TM^ SonicBone system—for predicting somatic maturation in youth athletes. **Methods**: The participants were female members of the Croatian national volleyball youth team (U-17) (*n* = 16). The study involved comprehensive measurements, including height, weight, sitting height, leg length, wrist diameter, hand joint diameter, hand grip strength, and ultrasound measurements for skeletal age assessment. **Results**: Correlation analysis showed moderate to strong correlations between the Moore and Mirwald skeletal age estimates, but both showed weaker correlations with the BAUSport^TM^ skeletal age. Repeated-measures ANOVA showed no significant difference between the Moore and Mirwald methods (*p* > 0.05); significant differences between both the Moore and Mirwald methods and the BAUSport^TM^ method (*p* < 0.05). Regression analysis revealed that height, weight, sitting height, leg length, wrist diameter, and hand joint diameter explained 69% of BAUSport^TM^, with wrist diameter being the only significant predictor. While the Moore and Mirwald methods remain useful tools for estimating the timing of an athlete’s growth spurt, BAUSport^TM^ represents a potential advancement in skeletal age assessment. Further research is needed to validate BAUSport^TM^ across diverse populations and optimize its calibration to accommodate anatomical variations. **Conclusions**: The findings suggest that with further refinement, BAUSport^TM^ could become a new standard for monitoring skeletal development in youth athletes. Additionally, studies should explore comparative analyses with other emerging technologies, such as genetic markers, hormonal assessments, and MRI, for further understanding of biological maturation in talent identification.

## 1. Introduction

The development of national youth teams is a critical component of talent identification in sports, as it sets the foundation for future athletic success. A key aspect of this process is understanding how biological maturation intersects with the selection of athletes. Considering biological maturation in talent selection processes is crucial to avoid disadvantaging late-maturing athletes and potentially reduce the relative age effect in sports [1]. Athletes who experience later maturation may face disadvantages in selection processes due to temporarily lower physical development compared to their earlier-maturing peers. In youth sports, coaches and evaluators often favor athletes with greater strength, speed, and height—traits typically associated with those who have entered puberty earlier. Without considering biological maturation, late-maturing athletes may be unfairly excluded despite their high long-term potential. As a result, promising talent may be overlooked, potentially hindering future excellence once full maturity is achieved. In addition to the challenges faced by late-maturing athletes, it is important to note that early maturation can offer benefits, such as improved initial athletic performance. However, while early maturing athletes may have an advantage in the short term, this can sometimes result in a plateau in development once their peers catch up in terms of maturity, highlighting the importance of considering maturation in long-term talent identification. By identifying an athlete’s maturity stage, performance evaluations can be adjusted accordingly, ensuring a more accurate and equitable selection process. Current scientific research on biological maturation in young athletes employs a variety of methodologies for its assessment that reflect the non-linear nature of the process. These methods range from non-invasive anthropometric approaches, such as predicting maturation status with height or secondary sexual characteristics, to imaging and biomarker analysis. While each of these approaches has its strengths and is continually evolving, no single method has been considered the gold standard, and there are known sex-, age-, and population-specific variations.

The Mirwald equation predicts years from peak height velocity using anthropometric measurements, showing high reliability in cross-validation studies [2]. However, while widely used, it has been shown to misclassify a significant portion of athletes [3]. Additionally, anthropometric measures have been used to develop prediction models for somatic maturity. Moore and colleagues refined existing equations, demonstrating good fit and calibration in external samples [4]. These models provide alternatives to commonly used methods and can be applied without specialized equipment. Various equations have been developed to predict maturity status, including those by Mirwald and Moore, which have demonstrated varying degrees of accuracy in Chilean children [5]. Fransen and colleagues developed an improved equation for estimating age at peak height velocity (APHV) using a maturity ratio, which showed better accuracy than previous models for both general and athletic populations [6]. Moraes Macêdo and colleagues created equations to predict skeletal age and sexual maturation index using anthropometric measurements in Brazilian children [7]. Malina and associates validated a non-invasive maturity estimate based on the percentage of predicted mature height against skeletal age in youth football players, finding moderate concordance between the two methods [8]. These studies demonstrate the ongoing efforts to develop and refine non-invasive techniques for assessing biological maturity, which can be valuable for talent identification and development in sports, as well as for medical diagnostics and disease prevention. Visual evaluation of individual growth curves demonstrated the highest concordance (≈80%) with maturity status classifications based on longitudinal data [3]. The percentage of predicted adult height method using Khamis–Roche or Tanner–Whitehouse 2 equations provides a reasonably valid alternative to maturity offset prediction equations, which tend to misclassify players [3].

Skeletal maturity refers to the development and maturation of bones, which can be assessed through methods such as bone age estimation. It is an important indicator of an athlete’s physical development and maturity stage, as it directly influences body mass, strength, flexibility, and cardiorespiratory fitness [9]. In sports performance, skeletal maturity can significantly affect an athlete’s abilities, particularly during periods of rapid growth, as those with more advanced skeletal maturity may have an advantage in strength and power. Understanding skeletal maturity helps in adjusting training loads, preventing injuries, and optimizing performance, which is critical for effective talent identification and long-term development in sports. Skeletal age assessment is crucial for evaluating growth, predicting final height, and guiding talent selection in youth sports [10]. While magnetic resonance imaging (MRI) is considered the gold standard, more pragmatic and cost-effective alternatives have been explored [11]. Ultrasound-based methods, such as BAUSport^TM^ SonicBone, have shown a high correlation with traditional radiographic techniques like the Fels method [12]. Various ultrasound imaging methods for assessing biological maturity have been developed, with the hand and wrist being the most commonly analyzed regions [13]. These methods generally demonstrate high reliability, but require further development to become a new gold standard. Understanding maturity-related changes is essential for managing training load, injury risk, and physical performance in youth soccer, particularly around the age of peak height velocity [14]. Quantitative ultrasound (QUS) techniques, such as the BAUSport^TM^ SonicBone device, have shown comparable results to traditional X-ray-based methods for skeletal age assessment [15]. Similarly, broadband ultrasonic attenuation (BUA) measurements of the calcaneus have been used to evaluate skeletal maturation in Japanese youth [16]. Such complementary non-invasive methods might provide advantages through reduced exposure to ionizing radiation when assessing particularly pediatric populations. Ultrasonographic techniques are also relatively inexpensive and readily available, and can be repeated for longitudinal studies. Additionally, they enable real-time imaging and dynamic evaluations, yielding detailed information on growth plate development and other biological maturation indicators without requiring specialized radiological infrastructure. Similarly, the Sunlight BonAge ultrasound device demonstrated good correlations with radiographic methods, offering a non-invasive and quick assessment for children aged 5–15 years [17]. Automated simplifications of the Eklof and Ringertz method, analyzing 3–5 ossification centers in carpal images, have shown high agreement with classical methods and offer a reliable, fast, and objective approach to skeletal age estimation [18].

Until now, no broad-scale studies have been conducted comparing ultrasound methods with traditional anthropometric methods at height, sitting height, and weight within algorithmic prediction. This study attempts to illustrate two different methodological approaches to obtain better information on the accuracy and applicability of various methods in sports selection and talent development contexts. The importance of accurately assessing the maturation of high-level athletes, particularly members of national teams, is crucial for ensuring fair competition, optimizing training regimens, and guiding future athletic development. National team athletes represent the pinnacle of talent, and their maturation status can have significant implications for long-term performance and injury prevention. This makes it all the more critical to accurately measure and track their progress, since the selection process for elite sports often favors athletes with specific physical and maturational characteristics, particularly in aesthetic sports [19]. The main aim of the study was to compare and evaluate three distinct protocols—Moor, Mirwald, and BAUSport^TM^—in terms of their methodologies, accuracy, and practical applications in predicting somatic maturation in youth athletes. The study seeks to understand how these methodologies correlate with each other and their practical implications in the fields of sports science and youth talent detection and athletic development.

## 2. Materials and Methods

The overall sample consisted of 16 female Croatian national volleyball youth team members (U-17), aged between 14 and 16 years (mean ± SD = 15.89 ± 0.58 years). All participants were of Caucasian origin and were actively involved in competitive volleyball at the national level. The participants were selected based on their membership in the Croatian national volleyball team, with all having a minimum of 4 years of training experience in the sport. However, no specific inclusion or exclusion criteria were applied beyond their national team status and training background. The selection was made by the national team coach and technical staff based on the players’ performances observed in their club games. All players were pre-selected by national-level scouts and coaching staff based on their technical and tactical performance, as well as club-level match assessments. This ensured a homogenous, high-performance sample representative of elite youth volleyball athletes. On average, participants trained 5 times per week. The inclusion criteria for this study were age and national team membership. Ethical approval for the study was obtained from the University of Split, Faculty of Kinesiology Research Ethics Board (003-08/20-04/00121818-205-02-05-20-006), and informed consent was provided by both the participants and their legal guardians.

The sample of variables was composed of chronological age, training experience, height (cm), weight (kg), sitting height (cm), leg length (cm), wrist diameter (cm), hand joint diameter (cm), hand grip strength (kg), and ultrasound measurements for skeletal age assessment. All measurements were performed by the same investigator who had extensive training and experience, and each measure was repeated three times, with the average used for statistical analysis. The participants wore the same training attire (shorts, shirt, socks) and removed footwear for all measures. All tests were carried out between 8:00 a.m. and 10:00 a.m. in a standardized indoor laboratory setting to minimize circadian variation.

Body weight (MC-780, Tanita Corporation, Tokyo, Japan) was recorded with electronic scales to the nearest 0.1 kg. Height was recorded to the nearest 0.1 cm using a portable stadiometer (Holtain, Harpenden, UK). Sitting height was assessed as the distance from the vertex to the base sitting surface. Results were recorded to the nearest 0.1 cm using a sitting height stadiometer (SHstad; Harpenden Sitting Table, Holtain, Harpenden, UK). The leg length was measured in a standing position, without shoes, with the feet slightly apart. The distance between the anterior iliac crest (upper part of the hip bone) and the medial malleolus (bony protrusion on the inner side of the ankle) was measured using a Martin Anthropometer. The result was displayed with a precision of 0.1 cm. Wrist and hand joint diameters were recorded to the nearest 0.01 cm using a sliding caliper (GPM Martin type Sliding Caliper, Bachenbülach, Switzerland). Hand diameter was measured across the metacarpophalangeal joints and wrist diameter across the styloid processes of the radius and ulna. All mentioned measures were obtained in one day. All anthropometric measurements were repeated three times, and average results were taken for statistical purposes. The grip strength of the dominant hand was measured using a standard adjustable digital hand grip dynamometer (Takei Scientific Instruments Co., Ltd., Tokyo, Japan) at standing position with shoulder adducted and neutrally rotated and elbow in full extension. The subjects were asked to put maximum force on the dynamometer thrice. The test was repeated three times, with adequate rest between attempts to avoid fatigue (1 min). The maximal value was recorded in kilograms.

Collected anthropological data were inserted into two algorithms to estimate three variables: age at peak height velocity (APHV), maturity offset (OFFSET) (measures the time from the peak height velocity), and skeletal age for each method (Mirwald and Moore method).

**Figure 1:**
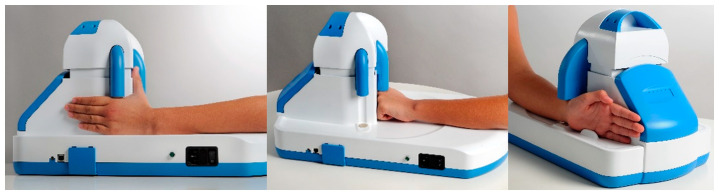
BAUSport^TM^ SonicBone device protocol (1st, 2nd and 3rd hand measurements positions) (source: https://sonicbonemedical.com/product/, accessed on 30 January 2025).

The BAUSport^TM^ SonicBone instrument system with accompanying software, produced by SonicBone Medical Ltd., Rishon LeZion, Israel, was used to estimate skeletal age (BAUSport^TM^ Skeletal Age) based upon ultrasound assessment of three skeletal locations on the left hand-wrist. Assessments were conducted by a professional who was trained in the use of the BAUSport^TM^ device. BAUSport^TM^ system device was placed on a stable table to avoid vibration or displacement during the test. The participants’ chair height was adjusted to ensure natural and comfortable hand placement. Participants removed all jewelry, rings, bracelets, watches, etc., before the measurement. A washable marker was used to mark the contact spots on the patient’s wrist and metacarpal sites. An even layer of ultrasound gel was applied at each designed location on the hand to establish acoustic contact between the ultrasound probes and the hand. During the measurement, the participants’ elbow touched the top side of the device surface. The angle between the arm and hand during measurement was between 130 and 140 degrees, and the hand was placed parallel to the device. In order to obtain a valid measurement result, all three measurements were performed in the following order: 1—wrist, 2—phalanx, 3—metacarpal (Figure 1). Information, based upon the speed at which high-frequency waves of an ultrasound pulse propagate through bone and distance attenuation factors (i.e., decay rate), is fed into an integrated algorithm using the scoring method designed by Tanner and Whitehouse [20]. The algorithm then provides an estimate of skeletal age and future adult stature. The time durations for the scans at each of the various sites were 12 s for the radius and ulna and 4 s for the proximal phalange and distal metacarpal. Total time for completing the assessment was approximately five to ten minutes per participant. The BAUSport^TM^ system has previously demonstrated high levels of repeatability and validity in young athletes and in the general population [12,15,21]. However, one of the technical limitations of the BAUSport™ device is that its skeletal age estimates do not fully align with traditional radiographic methods, such as the Fels method [12]. While it provides valuable non-invasive measurements, there can be fixed biases between the BAUSport™ estimates and radiographic assessments, leading to discrepancies in some cases.

A comparison of the methodological approach of the three maturation assessment methods is presented in Table 1.

Descriptive anthropometric parameters were calculated in the first step to provide an overview of the data, including means, standard deviations, and ranges (minimum and maximum values) for all variables, as well as the percentages of late, on-time, and early maturers (%). Secondly, using a paired samples *t*-test, the difference between the chronological and skeletal age of the participants was calculated. The third step contained correlation analyses with the aim of assessing the relationships between the skeletal age estimates of the three methods (Moore, Mirwald, and BAUSport^TM^), as well as their relationships with other measured parameters. In the fourth step, the authors investigated the results of repeated-measures analysis of variance (ANOVA) with a post hoc Tukey test to compare the skeletal age estimates from each pair of methods (Moore vs. Mirwald, Moore vs. BAUSport^TM^, Mirwald vs. BAUSport^TM^) to test for significant differences. Regression analysis was conducted to explore the predictive power of BAUSport^TM^ method and to assess the influence of other variables like height, weight, sitting height, leg length, hand grip strength, wrist, and hand joint diameter. Normality of data was assessed using the Shapiro–Wilk test prior to parametric statistical procedures. All analyses were conducted using Statistica 14.1, TIBCO Software Inc., Santa Clara, CA, USA.

## 3. Results

The best Croatian young female volleyball players, members of the Croatian U-17 national team, were tested for this research, and their results are presented in the tables below. Table 2 comprises the results of descriptive statistics for all applied variables (mean values, standard deviations, minimal and maximal results). The assumption of normality was verified using the Shapiro–Wilk test, which yielded non-significant results (*p* > 0.05) across all variables, indicating an approximately normal distribution suitable for parametric testing. The results presented show that the average age of the U-17 players was just under 16 years, while the youngest representative was only 14.7 years old. For comparison, the skeletal age of the participants was numerically higher, averaging 17.29 years. The results of the Moore and Mirwald OFFSET and APHV are basically aligned, and a relatively small standard deviation suggests homogeneity within the sample regarding maturity progression. The range for APHV is minimal, demonstrating a tightly clustered distribution, meaning that the cohort reached PHV around a similar age. However, the upper limits of skeletal age, for both the Moore and the Mirwald method, nearing almost 20 years suggests some advanced maturation cases. The values of skeletal age measured with the use of BAUSport^TM^ device showed somewhat lower values for both mean and minimal and maximal results.

The paired samples *t*-test revealed that the difference between chronological and skeletal age (measured with BAUSport^TM^) is significant (t = 5.31, *p* = 0.00).

**Figure 2:**
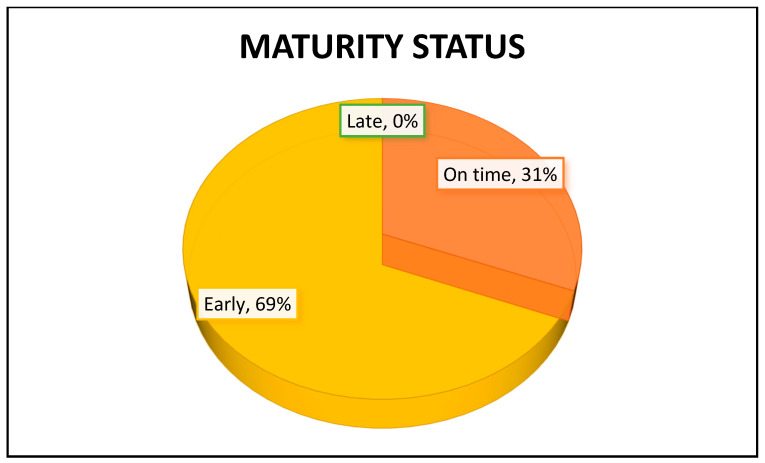
The percentages of late, on-time, and early maturers (%) in Croatian female national volleyball youth team members (U-16) according to the BAUSport^TM^ method.

For the BAUSport^TM^ measurement method (SonicBone ultrasound device), the maturation time for each individual (i.e., early, on time, late) is defined by the difference between their skeletal age and chronological age at the time of assessment and is presented in years. An individual with a skeletal age greater than their chronological age is considered advanced in maturation for their gender and age. Conversely, an individual with a skeletal age lower than their chronological age is considered delayed in maturation. When skeletal age and chronological age are equal, the individual is considered on time in maturation. In the case of the sample of young female volleyball players, it can be determined from Figure 2 that there were no participants with delayed maturation (those with a chronological age greater than their skeletal age). Only 31% of the participants were in the on-time maturation status, while as many as 69% were in the early stages of maturation.

**Figure 3:**
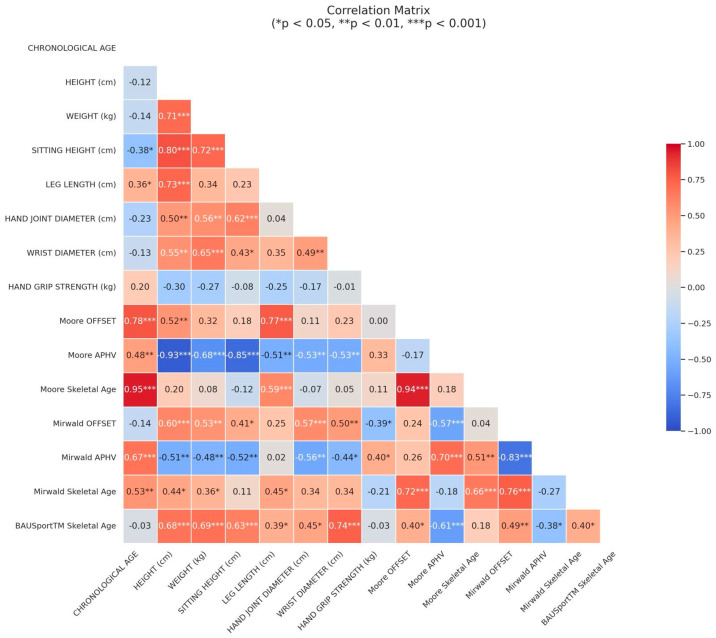
“The Heatmap” of the correlation matrix (the colors indicate the strength of the correlation, where warmer colors (red-orange) denote a positive correlation, and cooler colors (blue) denote a negative correlation). The numerical values inside the squares represent the actual correlation coefficients.

The correlation analysis drawn from the skeletal age assessment data in Figure 3 brings out several pertinent observations. Between the skeletal age estimates, the Moore and Mirwald methods show a moderate to strong correlation (r = 0.66), which implies an adequate agreement level regarding their assessments of somatic maturity. On the other hand, both methods exhibit weaker correlations with the BAUSport^TM^ skeletal age (Moore: r = 0.18; Mirwald: r = 0.4), indicating that they might be based on different criteria or scales for measuring skeletal development. Height, weight, and other physical measurements show varied degrees of correlation with skeletal age estimates. It is worth highlighting the significant correlation between wrist diameter and BAUSport^TM^ skeletal age (r = 0.74). The strong correlation implies that wrist diameter could be a reliable predictor of skeletal age.

As expected, results of repeated-measures ANOVA with post hoc Tukey test in Table 3 show that there is no significant difference between the Moore and Mirwald methods in estimating skeletal age (*p* > 0.05). Also, the results show that there is a significant difference between the Moore and Mirwald methods and the BAUSport^TM^ method in estimating skeletal age (*p* < 0.05). The Cohen’s d values for the post hoc pairwise comparisons showed a small difference between the Moore and Mirwald methods (d = 0.12), while the differences between Moore and BAUSport^TM^ (d = 1.35) and Mirwald and BAUSport^TM^ (d = 1.33) were large, indicating significant differences between these methods.

The regression analysis from Table 4 shows that height, weight, sitting height, leg length, hand joint, and wrist diameter explained 69% of BAUSport^TM^ skeletal age estimation, with wrist diameter being the only significant predictor.

## 4. Discussion

The U17 national team serves as a platform for developing the most talented young volleyball players in the country. They are selected from domestic clubs and gather for training camps and tournaments. Since this team precedes the elite senior national team, it is extremely important to monitor the characteristics, abilities, and biological age of the players to ensure the proper development and progress of each individual athlete. This allows for an individualized approach to training, load adjustment, and injury prevention, thereby maximizing the potential for success at the highest level of competition. Proper monitoring of biological age helps in understanding the physical development of the players, ensuring that they develop optimally and are prepared for the challenges of senior competition. In this context, the data contribute to understanding skeletal development patterns within a highly homogenous group of young athletes, with implications for training, talent identification, and health monitoring. A sample of elite Croatian young volleyball players demonstrated a tendency toward early maturation, as the difference between chronological and biological age proved to be significant. One of the key aspects of this finding is the accelerated biological development of volleyball players, which indicates early pubertal maturity. This phenomenon has already been documented in previous research showing that female athletes, especially in sports requiring high levels of physical performance, may exhibit advancements in biological development compared to their less physically active peers [22]. Similar findings were reported on male volleyball players, identifying how young volleyball players classified as “early” seemed to show anthropometric characteristics linked to better performance at the tournament (higher height, upper arm and calf muscle area, fat mass percentage, and total fat-free mass) [23]. To further explore the risk of bias in talent selection, it is essential to recognize that the preference for early-maturing athletes may inadvertently disadvantage those with later maturation, who may not yet exhibit their full potential. To mitigate this bias, talent identification systems should consider not only physical attributes but also the long-term development trajectory, incorporating measures to assess athletes’ maturity stages and accounting for the potential advantages of late-maturing athletes in the future.

Accelerated biological development may be associated with various factors, including genetic predisposition, physical activity levels, nutrition, and exposure to stress. It is important to note that although advanced skeletal age may enable earlier participation in competitions with older athletes, it also carries certain risks, such as an increased likelihood of injuries due to earlier closure of growth plates (epiphyseal plates) and excessive stress on joints and bones [24]. Further, early-maturing athletes may be more susceptible to overtraining due to their accelerated physical development and increased training intensity. This can lead to an imbalance between training load and recovery, increasing the risk of injury and hindering long-term performance progress. Overtraining can increase the risk of early burnout due to higher physical demands and psychological pressure. Athletes who mature early may push themselves too hard, leading to fatigue, and potential long-term performance declines. The difference between chronological and skeletal age can significantly impact how these athletes are trained and monitored. Since biologically older athletes may exhibit higher levels of physical strength, endurance, and explosiveness, coaches might be inclined to increase training intensity to capitalize on these advantages. However, it is crucial to ensure that training is tailored to the individual needs of athletes, considering not only their physical capacities but also their long-term health.

This is particularly important during the period of accelerated growth (“peak height velocity”), which occurs at different times for each individual. Unsurprisingly, the risk of injury significantly increases during the most intense growth periods [25,26], so coaches must exercise particular caution in planning the training process for growing athletes. One strategy may include regular monitoring of skeletal age and other indicators of biological development to optimize training methods and reduce injury risk. For instance, research suggests that high-intensity training should be adjusted according to the degree of biological maturity, allowing for long-term sports development without compromising health [27]. In addition to monitoring immediate performance, it is crucial to adopt a long-term perspective when evaluating an athlete’s career. While short-term success may be a motivating factor, focusing solely on immediate results can overlook the potential for long-term development and sustainability in an athlete’s career. Taking a more holistic approach to training and progression, considering maturation status and long-term goals, helps ensure that athletes reach their full potential without prematurely peaking or risking injury.

These results show an interesting and potentially significant pattern in the maturation of young female volleyball players. First, the fact that 69% of the participants were classified in the early maturation stage may indicate that physical development plays a key role in the selection of young volleyball players at the national level. Early maturation is associated with faster physical development, including growth in height, strength, and muscle mass, all of which are key components of success in volleyball. These results may reflect the tendency of selectors to choose players who are physically superior to their peers, which can have a direct impact on their on-court performance.

Second, only 31% of the participants were in the on-time maturation status, which is a relatively low percentage compared to expected distributions in the general population. These data may suggest that girls who mature in accordance with their chronological age may be at a disadvantage in the selection process for top-level sports teams, as their peers with earlier maturation may physically outmatch them. In the context of long-term development, it is important to consider the implications of these results. Early maturation may bring certain advantages in the earlier stages of a career but can also lead to early burnout, increased injury risk, and a decline in long-term sports performance [28]. These risks are especially pronounced in athletes who specialize early in one sport, which can result in overtraining and increased stress on the young body. Therefore, it would be useful to investigate how early maturation affects the careers of these athletes and whether there is a need to adjust selection criteria to ensure the long-term sustainability and success of young volleyball players.

The Moore and Mirwald methods for assessing skeletal age and somatic maturation are based on the concept of predicting APHV. These methods utilize growth curves that plot the anthropometric measurements over time. However, like all predictive methods, they have limitations. The accuracy of these methods can be influenced by genetic, nutritional, and environmental factors, and they may not be as precise as other more technologically advanced methods [3]. Also, the authors [2] caution against using maturity offset as a continuous measure, and instead recommend considering it as a categorical variable.

On the other hand, the BAUSport^TM^ method is a more recent and technologically advanced approach for assessing skeletal age and somatic maturation, particularly in youth athletes. Unlike the Moore and Mirwald methods, which rely on anthropometric measurements and growth curves, the BAUSport^TM^ method utilizes ultrasound technology to evaluate skeletal age. The technology measures parameters such as the speed of sound (SOS) and the distance attenuation factor (ATN) through the bone. Studies have shown that the BAUSport^TM^ method has high repeatability and reliability [12,15,21]. It has been found to be comparable to traditional X-ray-based methods in terms of accuracy in skeletal age assessments. While the BAUSport^TM^ method is still under development and refinement, particularly in terms of reproducibility and eliminating confounding factors, it shows promising results, as many recent studies used this technology for assessing skeletal maturity purposes [11,21]. Despite these promising aspects, further validation of the measurement process is necessary. In our study, we encountered a case where the BAUSport™ device was unable to obtain a measurement for one participant, displaying an error message instead. Our hypothesis is that this issue may have been caused by the participant’s unusually large hand size, specifically the diameter of the wrist joint or the thickness of the metacarpal region, which may have exceeded the device’s measurement range. Due to the inability to obtain a valid measurement, this participant had to be excluded from the study. This limitation underscores the need for manufacturers to investigate the root causes of such errors and assess whether the device’s sensor calibration and measurement algorithm can be optimized to accommodate a wider range of hand dimensions. Future research should explore whether adjustments in sensor sensitivity, hardware design, or software parameters could improve measurement reliability across diverse anatomical variations. A potential extension of the BAUSport™ device could involve adapting its calibration and measurement algorithms to assess skeletal age beyond the limits of youth athletes, enabling its application in longevity research and aging studies by evaluating bone health, density, and structural changes over time in adult and elderly populations.

The strongly positive correlation between the Moore and Mirwald methods indicates a high agreement level in their estimates of skeletal age, which was expected. Both methods rely on somatic indicators and growth variables, which is probably the reason for their correlation. This confirms their suitability for assessing skeletal maturity, especially in homogeneous groups. However, methodological similarities between the two may hinder their ability to detect subtleties of skeletal development, particularly when maturation is advanced or delayed. The weaker correlations between the skeletal age indicated by BAUSport™ and those derived from the Moore and Mirwald methods imply that BAUSport™ utilizes different standards or scales in evaluating skeletal maturity. This difference lies in the fact that BAUSport™ relies on a direct assessment of bone using ultrasound technology. Although this raises questions about the comparability of methods, it also opens up the possibility that BAUSport™ may provide valuable information on skeletal development that somatic methods may fail to detect. The lower skeletal age values observed in the BAUSport^TM^ method may reflect the device’s sensitivity to structural bone maturity rather than somatic development proxies, underscoring its potential for the early detection of maturation-related injury risk.

There is a remarkable correlation between the diameter of the wrist and BAUSport™ skeletal age. This makes for a good indicator of skeletal maturity since, probably, it is involved with an area of bone development that correlates directly with both chronological and biological aging. The regression analysis further confirmed the influence of the measured variables on the skeletal age measured by BAUSport^TM^, but with wrist diameter standing out as the only significant predictor. This may be because measures such as height, weight, leg length, and sitting height are more influenced by somatic growth, while the BAUSport™ method accurately tracks the development of specific bone structures. These observations point to an idea that could be related to the results from a study using a novel method of wrist skeletal maturity [29]. The authors used epiphyseal–metaphyseal ratios of the first and third metacarpals, combined with chronological age and sex, and showed improved accuracy in estimating skeletal maturity compared to the Greulich and Pyle technique, especially in preadolescents. Such results could also lead to the fact that the wrist is an important factor in maturity estimations. These results further confirm that skeletal age assessment requires a focus on specific anatomical indicators rather than general somatic measurements. Therefore, this supports the idea that making measurements of wrist diameter is a workable, non-invasive approach to estimating skeletal age in situations where there may not be access to sophisticated imaging equipment like BAUSport^TM^. On the other hand, BAUSport™ SonicBone technology is a valuable tool for skeletal age assessment in children. Skeletal growth has critical phases and the BAUSport^TM^ technology can determine these phases during periods of growth, like the “dangerous zone”, during which injuries are more common. Children are usually at a greater risk of skeletal injuries during a growth spurt, as they develop rapidly [24,30]. The danger of injury in this phase is what makes it the dangerous zone. By accurate assessment of skeletal maturity, the BAUSport^TM^ system allows practitioners to track growth and development, such as when certain individuals are prone to injuries and when specific training can be incorporated. The injuries are a result of overtraining, so early detection of the disabling illness can aid prevention. Skeletal age assessment using BAUSport™ SonicBone technology is extremely precise and safe, but should still be combined with anthropological methods like Moore’s and Mirwald’s methods. Their distinct methods of determining skeletal maturity and the varying standards they use result in different outcomes, which cause disparity and confusion in practice. The integration of BAUSport^TM^ technology and anthropological methods provides a more nuanced perspective on the growth and development of an athlete, including skeletal age.

Further studies are needed, though, to determine if it is consistent across different populations and age groups. Also, research should explore the impact of genetic, nutritional, and environmental factors on the correlation between anthropometric measures and skeletal age. One of the most effective ways to investigate genetic factors would be through twin studies, which allow for the comparison of monozygotic and dizygotic twins to distinguish genetic influences from environmental effects on the relationship between anthropometric measures and skeletal age. Researchers could incorporate dental age assessment as an additional indicator of biological age, exploring its potential relationship with skeletal age and building on previous studies that have confirmed the connection between these two parameters [31]. Finally, validation of the model on longitudinal samples is necessary to determine its reliability over time.

## 5. Conclusions

The findings indicate that 69% of the national team members, with zero late participants, were early maturers, suggesting a potential selection bias favoring physically advanced athletes, which may overlook late-maturing individuals with long-term athletic potential. This highlights the need for a more holistic talent identification approach that considers biological maturation alongside skill development and long-term athletic progression to prevent premature exclusion of late bloomers.

In terms of practical implications, coaches, sports doctors, and selectors should continuously monitor biological maturation to ensure that athletes’ development is optimized in accordance with their individual growth. This includes being mindful of the risks of early maturation, such as the potential for overtraining, early burnout, and growth-related injuries.

The study confirms that while the Moore and Mirwald methods are traditional and useful tools for estimating the timing of a youth athlete’s growth spurt, the BAUSport^TM^ method represents a significant advancement in this field. This method offers a non-invasive, safe, fast, and reliable alternative for effectively monitoring the maturation of young athletes. The observed limitations in applying the BAUSport™ method to a subset of participants emphasize the need for further validation of BAUSport™ ultrasound-based techniques across diverse populations to ensure broader applicability and methodological reliability.

The three methods used in this study have different approaches to maturity assessment:

- The Moore and Mirwald methods are simple, quick, and easy to apply, but they do not directly measure skeletal age, relying instead on statistical models.
- The BAUSport^TM^ method uses direct ultrasound measurements, providing greater precision and reliability, but requires specialized equipment and further validation.

A combination of ultrasound methods and anthropometric measurements could improve the accuracy of skeletal maturation assessment and optimize talent selection. The main contribution of this research is demonstrating that while the Moore and Mirwald methods offer valuable insights for coaches and sports scientists, the BAUSport^TM^ method has the potential to become a new standardized method for monitoring the skeletal development of young athletes. These findings underscore the importance of integrating both anthropometric prediction models and direct skeletal assessment tools into longitudinal talent development strategies. Such a dual-modality approach may provide a more accurate, ethical, and individualized pathway for youth athlete monitoring and selection. Future studies should include comparative analyses with other advanced methods, such as genetic markers, hormonal assessments, or MRI technology, to further enhance the understanding of biological maturation and its role in sports talent development. Additionally, further investigation is needed to optimize the calibration of the BAUSport™ device to accommodate a wider range of anatomical variations and ensure its applicability across diverse populations.

## Figures and Tables

**Table 1:** Main methodological differences between the Moore, Mirwald, and BAUSport^TM^ methods.

Characteristic	Moore Method	Mirwald Method	BAUSport^TM^ Method
Type of measurement	Anthropometric	Anthropometric	Ultrasound
Main variables	Height, sitting height, leg length, weight, sex	Same as Moore	Ultrasound-based bone density, speed of sound through bone
Accuracy	Moderate	Moderate	High for skeletal age
Ease of application	Very simple	Very simple	Requires specialized equipment
Application in sports	Monitoring growth and predicting PHV	Same as Moore	Direct assessment of skeletal age and bone development
Main advantage	Improved accuracy compared to Mirwald	Simple and widely used method	Non-invasive alternative to X-ray-based skeletal age assessment
Main limitation	Relies on statistical estimations	May underestimate or overestimate maturation	Calibration issues and still uninvestigated sensitivity to anatomical variations

**Table 2:** Descriptive statistical parameters of all applied variables (Mean—mean value, SD—standard deviation, Min—minimal result, Max—maximal result).

	Mean	SD	Min	Max
Chronological age	15.89	0.58	14.71	16.49
Height (cm)	180.63	5.06	171.00	187.00
Weight (kg)	67.50	5.83	54.00	75.00
Sitting height (cm)	93.67	3.15	87.30	97.50
Leg length (cm)	86.68	3.12	81.70	92.30
Hand joint diameter (cm)	8.22	0.37	7.70	8.80
Wrist diameter (cm)	5.68	0.27	5.10	6.10
Hand grip strength (kg)	33.67	3.91	26.30	38.70
Moore OFFSET	3.65	0.52	2.83	4.41
Moore APHV	11.24	0.36	10.74	11.98
Moore Skeletal Age	18.55	1.03	16.54	19.76
Mirwald OFFSET	3.54	0.76	2.81	5.95
Mirwald APHV	11.36	1.02	7.91	12.44
Mirwald Skeletal Age	18.43	0.88	16.56	19.82
BAUSport^TM^ Skeletal Age	17.29	0.89	15.46	18.64

Legend: OFFSET—measures the time from the peak height velocity, APHV—age at peak height velocity.

**Table 3:** Repeated-measures ANOVA with post hoc Tukey test for skeletal age assessment using the Moore, Mirwald, and BAUSport^TM^ methods.

Comparison	Mean Difference	*p*-Value	Interpretation
Moore vs. Mirwald	0.115	0.935	no significant difference (*p* > 0.05)
Moore vs. BAUSport^TM^	−1.252	0.001	significant difference (*p* < 0.05)
Mirwald vs. BAUSport^TM^	−1.136	0.004	significant difference (*p* < 0.05)

**Table 4:** The regression analysis results for the BAUSport^TM^ method.

Method	Coefficients	
BAUSport^TM^	height: 0.035, weight: 0.027, sitting height: 0.017, leg length: 0.019, hand joint diameter: 0.048, wrist diameter: 1.626 *	R = 0.83 R^2^ = 0.69 *p* = 0.04

Legend: * significant predictor (*p* < 0.05).

## Data Availability

The data presented in this study are available upon request from the corresponding author.

## References

[1] Müller L., Müller E., Hildebrandt C., Kapelari K., Raschner C. (2015). The Assessment of Biological Maturation for Talent Selection—Which Method Can Be Used?. Sportverletz. Sportschaden.

[2] Mirwald R.L., Baxter-Jones A.D.G., Bailey D.A., Beunen G.P. (2002). An Assessment of Maturity from Anthropometric Measurements. Med. Sci. Sports Exerc..

[3] Monasterio X., Gil S.M., Bidaurrazaga-Letona I., Cumming S.P., Malina R.M., Williams S., Larruskain J. (2024). Estimating Maturity Status in Elite Youth Soccer Players: Evaluation of Methods. Med. Sci. Sports Exerc..

[4] Moore S.A., McKay H.A., Macdonald H., Nettlefold L., Baxter-Jones A.D., Cameron N., Brasher P.M. (2015). Enhancing a Somatic Maturity Prediction Model. Med. Sci. Sports Exerc..

[5] Cossio-Bolaños M., Vidal-Espinoza R., de Campos L.F.C.C., Sulla-Torres J., Cossio-Bolaños W., Albornoz C.U., Gómez-Campos R. (2021). Equations Predicting Maturity Status: Validation in a Cross-Sectional Sample to Assess Physical Growth and Body Adiposity in Chilean Children and Adolescents. Endocrinol. Diabetes Nutr..

[6] Fransen J., Bush S., Woodcock S., Novak A., Deprez D., Baxter-Jones A.D., Lenoir M. (2018). Improving the Prediction of Maturity from Anthropometric Variables Using a Maturity Ratio. Pediatr. Exerc. Sci..

[7] Moraes Macêdo M., Linhares R.V., Filho J.F. (2015). Equations for Determining Bone Age and Sexual Maturation of Children and Adolescents. Rev. Salud Pública.

[8] Malina R.M., Dompier T.P., Powell J.W., Barron M.J., Moore M.T. (2007). Validation of a Noninvasive Maturity Estimate Relative to Skeletal Age in Youth Football Players. Clin. J. Sport Med..

[9] Gouvea M., Cyrino E.S., Ribeiro A.S., da Silva D.R.P., Ohara D., Valente-Dos-Santos J., Coelho-E-Silva M.J., Ronque E. (2016). Influence of skeletal maturity on size, function and sport-specific technical skills in youth soccer players. Int. J. Sports Med..

[10] Suh J., Heo J., Kim S.J., Park S., Jung M.K., Choi H.S., Choi Y., Oh J.S., Lee H.I., Lee M. (2023). Bone Age Estimation and Prediction of Final Adult Height Using Deep Learning. Yonsei Med. J..

[11] Leyhr D., Murr D., Basten L., Eichler K., Hauser T., Lüdin D., Romann M., Sardo G., Höner O. (2020). Biological Maturity Status in Elite Youth Soccer Players: A Comparison of Pragmatic Diagnostics with Magnetic Resonance Imaging. Front. Sports Act. Living.

[12] Cumming S., Pi-Rusiñol R., Rodas G., Drobnic F., Rogol A.D. (2023). The Validity of Automatic Methods for Estimating Skeletal Age in Young Athletes: A Comparison of the BAUSport Ultrasound System and BoneXpert with the Radiographic Method of Fels. Biol. Sport.

[13] Rüeger E., Hutmacher N., Eichelberger P., Löcherbach C., Albrecht S., Romann M. (2022). Ultrasound Imaging-Based Methods for Assessing Biological Maturity during Adolescence and Possible Application in Youth Sport: A Scoping Review. Children.

[14] Towlson C., Salter J., Ade J.D., Enright K., Harper L.D., Page R.M., Malone J.J. (2021). Maturity-Associated Considerations for Training Load, Injury Risk, and Physical Performance in Youth Soccer: One Size Does Not Fit All. J. Sport Health Sci..

[15] Rachmiel M., Naugolni L., Mazor-Aronovitch K., Koren-Morag N., Bistritzer T. (2017). Bone Age Assessments by Quantitative Ultrasound (SonicBone) and Hand X-ray Based Methods Are Comparable. Isr. Med. Assoc. J..

[16] Sasaki M., Motegi E., Soejima U., Nomura M., Kaneko Y., Shimizu T., Takeuchi F., Yamaguchi T., Yamanaka S., Yamaguchi H. (2003). Appraisal of Bone Maturity Age Derived from Broadband Ultrasonic Attenuation in Japanese Children and Adolescents. Bull. Tokyo Dent. Coll..

[17] Shimura N., Koyama S., Arisaka O., Imataka M., Sato K., Matsuura M. (2005). Assessment of Measurement of Children’s Bone Age Ultrasonically with Sunlight BonAge. Clin. Pediatr. Endocrinol..

[18] Olivete Júnior C., Rodrigues E.L.L. (2010). Bone Maturity: Estimation by Means of Eklof and Ringertz Method Simplifications. Radiol. Bras..

[19] Baxter-Jones A.D., Thompson A.M., Malina R.M. (2002). Growth and Maturation in Elite Young Female Athletes. Sports Med. Arthrosc. Rev..

[20] Cameron N. (1993). The Tanner-Whitehouse II Skeletal Maturity Method: Rationale and Applicability. Clin. Pediatr. Endocrinol..

[21] Ruf L., Cumming S., Härtel S., Hecksteden A., Drust B., Meyer T. (2021). Construct Validity of Age at Predicted Adult Height and BAUS Skeletal Age to Assess Biological Maturity in Academy Soccer. Ann. Hum. Biol..

[22] Malina R.M., Bouchard C., Bar-Or O. (2004). Growth, Maturation, and Physical Activity.

[23] Grigoletto A., Mauro M., Toselli S. (2023). Differences in Body Composition and Maturity Status in Young Male Volleyball Players of Different Levels. J. Funct. Morphol. Kinesiol..

[24] Caine D.J., Goodwin B.J. (2016). Risk Factors for Injury in Pediatric and Adolescent Sports. Injury in Pediatric and Adolescent Sports.

[25] Rumpf M.C., Cronin J. (2012). Injury Incidence, Body Site, and Severity in Soccer Players Aged 6–18 Years: Implications for Injury Prevention. Strength Cond. J..

[26] van der Sluis A., Elferink-Gemser M.T., Coelho-e-Silva M.J., Nijboer J.A., Brink M.S., Visscher C. (2014). Sport Injuries Aligned to Peak Height Velocity in Talented Pubertal Soccer Players. Int. J. Sports Med..

[27] McManus A.M., Armstrong N. (2011). Physiology of Elite Young Female Athletes. Med. Sport Sci..

[28] Whitney K.E., d’Hemecourt P.A., Stracciolini A. (2023). Youth Sport Specialization: Risks, Benefits, and Mental Health Considerations. Psychological Considerations in the Young Athlete.

[29] Huang L.F., Furdock R.J., Uli N., Liu R.W. (2022). Estimating Skeletal Maturity Using Wrist Radiographs during Preadolescence: The Epiphyseal: Metaphyseal Ratio. J. Pediatr. Orthop..

[30] Wik E.H., Martínez-Silván D., Farooq A., Cardinale M., Johnson A., Bahr R. (2020). Skeletal Maturation and Growth Rates Are Related to Bone and Growth Plate Injuries in Adolescent Athletics. Scand. J. Med. Sci. Sports.

[31] Bala M., Pathak A., Jain R.L. (2010). Assessment of Skeletal Age Using MP3 and Hand-Wrist Radiographs and Its Correlation with Dental and Chronological Ages in Children. J. Indian Soc. Pedod. Prev. Dent..

